# The approach of World Health Organization to articulate the role and assure impact of vaccines against antimicrobial resistance

**DOI:** 10.1080/21645515.2022.2145069

**Published:** 2022-11-24

**Authors:** Isabel Frost, Anand Balachandran, Sarah Paulin-Deschenaux, Hatim Sati, Mateusz Hasso-Agopsowicz

**Affiliations:** a Immunization, Vaccines & Biologicals, UHC/Life Course, World Health Organization, Geneva, Switzerland; b Imperial College London, London, UK; c Surveillance, Prevention and Control Department, AMR Division, World Health Organization, Geneva, Switzerland; d Global Coordination and Partnership Department, AMR Division, World Health Organization, Geneva, Switzerland

**Keywords:** Antimicrobial resistance, vaccines, value of vaccines

## Abstract

Antimicrobial resistance (AMR) is a growing global problem and there were an estimated 4.95 million deaths associated with bacterial AMR worldwide in 2019. Vaccines can impact AMR by preventing infections and reducing the need for antibiotics which will inadvertently slow the emergence of AMR. Effective infection prevention and control (IPC) has been identified as the cornerstone action to combat AMR by the World Health Assembly and the Global Action plan on AMR. Similarly, the Immunization Agenda 2030 highlights vaccines as critical tools to combat AMR. This article summarizes the strategy of the World Health Organization to understand, articulate and communicate the important role of vaccines in countering AMR. The work is organized around developing a strategy, understanding the pipeline of vaccines in development, articulating the value of vaccines against AMR, and assuring sustainable impact of vaccines at a country level to combat AMR.

Antimicrobial Resistance (AMR) occurs when bacteria, viruses, fungi and parasites no longer respond to antimicrobial agents. As a result of drug resistance, antibiotics and other antimicrobial agents become ineffective and infections become difficult or impossible to treat, increasing the risk of disease spread, severe illness and death. The prevalence of AMR is growing rapidly^1^ and pathogens resistant to all classes of antibiotics have been reported more frequently in recent years.^2^ As a consequence, many resistant infections are becoming more difficult to treat resulting in health, economic and societal loss. In 2019, 4.95 million deaths were associated with drug-resistant bacterial infections globally.^3^ The antimicrobial resistant infections are more expensive to treat, are associated with higher mortality and morbidity rates as well as high socioeconomic impact. Estimates suggest that 28 million people will fall into poverty worldwide due to AMR with an increase in health-care costs of up to US$1 trillion worldwide by 2050,^4^ with low-income countries expected to be the most impacted. A rapid and multifaceted response is needed to prevent the significant disease burden and socio-economic cost.^5^

The Global Action Plan on AMR lists five strategic objectives to contain AMR: optimizing the use of antimicrobials, preventing infections (including the use of vaccines), strengthening surveillance and research, improving awareness and understanding of AMR, and investing in new medical products.^6^

The Global Action Plan lays the blueprint for countries to develop country-specific National Action Plans on AMR. Vaccines play an important role in preventing infections (drug-susceptible and drug-resistant), and reducing^7,8^ antibiotic use, a key driver of AMR.^9,10^ Vaccines therefore can contribute to reducing selection for AMR in both the target pathogen (for bacterial vaccines) as well as in bacterial species that are not directly targeted by the used antibiotics (bystander effect) (Figure 1). A study of a pneumococcal conjugate vaccine (PCV) in South Africa observed a 67% reduction in penicillin-resistant invasive disease in the PCV-vaccinated group,^11^ and post-licensure PCV studies observe near elimination of resistant strains.^12^ Estimates suggest that pneumococcal and rotavirus vaccines prevent 23.8 million and 13.6 million episodes of antibiotic-treated illness, respectively, among children under five years of age in LMICs each year.^10^ Similarly, influenza vaccine has been shown to reduce days of antibiotic use among healthy adults, likely through reduction inappropriate empiric prescribing.^13^ Furthermore, typhoid conjugate vaccine, recently introduced to tackle drug-resistant typhoid in Pakistan, is expected to avert 895,000 of extensive drug resistant typhoid cases over the next ten years.^14,15^

**Figure 1.**
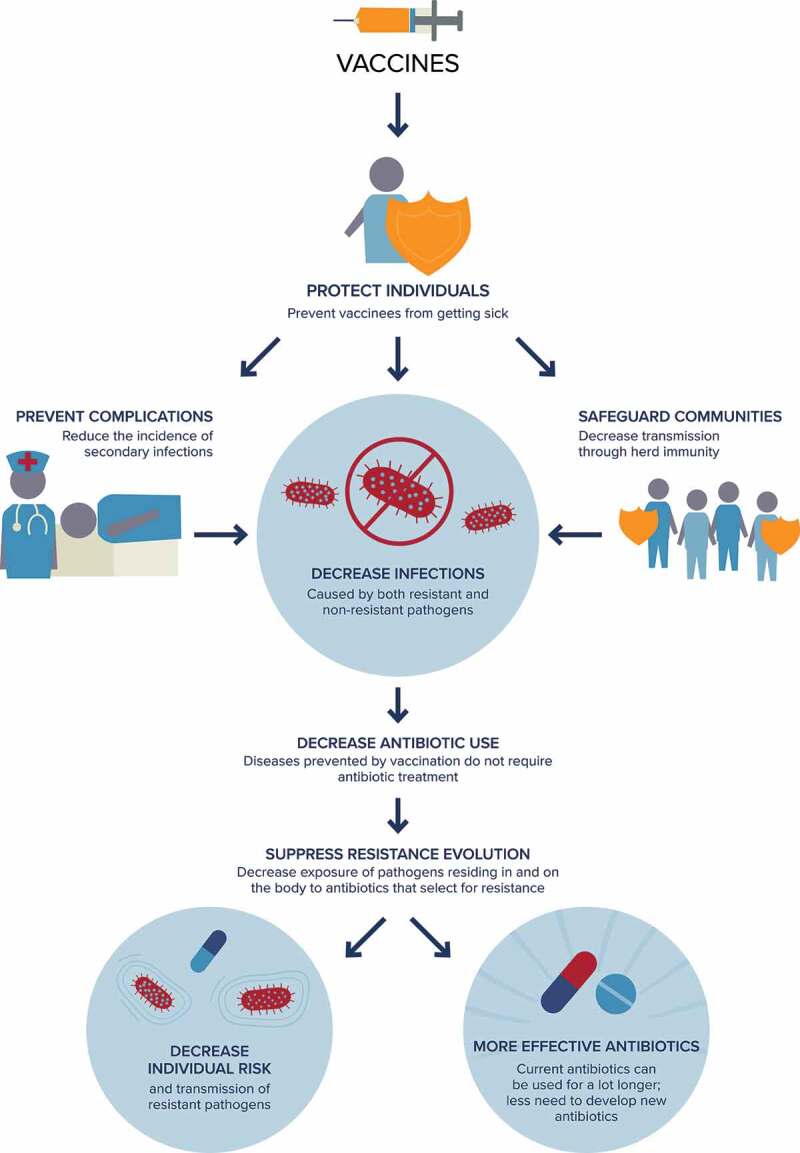
How do vaccines prevent AMR? An overview of mechanisms.

Immunization was highlighted as one of the tools to combat AMR in the Immunization Agenda 2030: A global strategy to leave no one behind.^16^ To develop a strategy on vaccines and AMR, the World Health Organization (WHO) has published an action framework^17,18^ - a technical annex to the Immunization Agenda 2030. The action framework describes a vision for vaccines to contribute fully, sustainably and equitably to the prevention and control of AMR by preventing infections and reducing antimicrobial use. To achieve this vision, the document articulates a list of priority actions to be taken by AMR and immunization stakeholders in three areas: expanding the use of licensed vaccines to maximize impact on AMR, developing new vaccines that contribute to the prevention and control of AMR, and expanding and sharing knowledge about the impact of vaccines on AMR (Panel 1). This document aims to support alignment of activities among international, regional and national vaccine and AMR implementing partners, and to structure and articulate key priority actions needed to articulate the value of vaccines against AMR and lead to implementation and impact.

**Panel 1.** Goals and objectives to leverage vaccines to prevent AMR.

*Goal 1. Expand the use of licensed vaccines to maximize impact on AMR* Objective 1. Increase coverage of vaccines with impact on AMR Objective 2. Update recommendations and normative guidance in both the vaccine and AMR sectors to include the role of vaccines to control AMR Objective 3. Improve awareness and understanding of the role of vaccines in limiting AMR through effective communication, education and training	
*Goal 2. Develop new vaccines that contribute to prevention and control of AMR* Objective 4. Bridge the funding gap for R&D of new vaccines with potential for global AMR impact Objective 5. Develop regulatory and policy mechanisms to accelerate approval and use of new vaccines that can reduce AMR	
*Goal 3. Expand and share knowledge of vaccine impact on AMR* Objective 6. Improve methodologies and increase collection and analysis of data to assess vaccine impact on AMR, including antimicrobial use Objective 7. Develop estimates of vaccine value to avert the full public health and socioeconomic burden of AMR	

There is growing recognition that vaccines are powerful tools to combat AMR. For example, the Wellcome Trust recommends to increase uptake of vaccines for *Salmonella* typhi, *Streptococcus pneumoniae*, and *Hemophilus influenzae tybe b*; and to bring to market vaccines for *Shigella*, non-typhoidal *Salmonella*, and enteric *Escherichia coli*.^8^ Gavi, the Vaccine Alliance analyzed the value of vaccines against AMR to inform their Vaccine Investment Strategy in 2018. They found that pneumococcal, typhoid and malaria vaccines have the highest value against AMR.^19^

The WHO has identified 12 priority pathogens for which new antibiotics are most urgently needed^20^ and has recently analyzed the preclinical and clinical pipeline for vaccines against these pathogens, in addition to *Clostridioides difficile* and *Mycobacterium tuberculosis*.^21^ The analysis builds on previous initiatives to evaluate the pipeline of antibacterials in preclinical^22^ and clinical^23,24^ development against the priority pathogens. In clinical development, 61 vaccine candidates were identified that target the priority pathogens, which were also classified into four groups (Figure 2). Group A, consisting of pathogens on the priority pathogen list for which vaccines already exist and includes *Salmonella enterica* ser. Typhi, *S. pneumoniae*, *Haemophilus influenzae* type b, and *M. tuberculosis*. Group B, consisting of pathogens with vaccines in advanced clinical development including extraintestinal pathogenic *Escherichia coli, Salmonella enterica* ser. Paratyphi A, *Neisseria gonorrhoeae*, and *C. difficile*. Group C, consisting of pathogens with vaccines in earlier phases of clinical development; enterotoxigenic *E. coli*, *Klebsiella pneumoniae*, non-typhoidal *Salmonella, Shigella* spp. and *Campylobacter* spp. Finally, Group D includes pathogens with either no candidates in clinical development or those assessed by expert consultations to have low development feasibility. These are *Pseudomonas aeruginosa*, *Acinetobacter baumannii*, *Staphylococcus aureus*, *Helicobacter pylori*, *Enterococcus faecium*, and *Enterobacter* spp. WHO published the analyses in a 2022 report (ref) calling for the rapid introduction and expansion of already existing vaccines at country level, acceleration of clinical trials for pathogens with vaccines in late-stage development, research to understand the value of vaccines against pathogens in early stages of clinical development, and alternative ways to tackle AMR for pathogens with no vaccines in clinical development.

**Figure 2.**
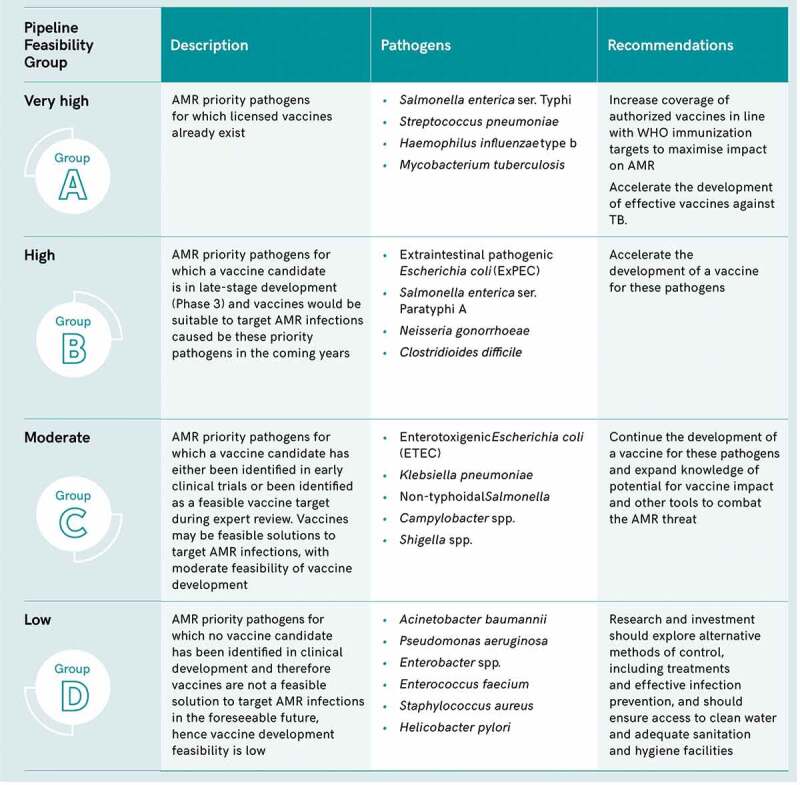
Summary of pipeline findings and recommendations for priority AMR pathogens.^21^.

Despite the aforementioned WHO, Wellcome Trust and Gavi reports and analyses, the impact of vaccines on AMR is often not incorporated into evaluations of vaccine value. This means vaccines are often undervalued in terms of their impact on AMR across the public health community. To systematically incorporate the impact of vaccines on AMR, a value attribution framework is needed to guide ways of articulating vaccine impact on AMR. To this end, WHO is developing a value attribution framework for the impact of vaccines on AMR. The document considers the value of vaccines for 30 pathogens including bacteria, viruses, fungi and parasites, across five criteria: (1) vaccine averted AMR health burden,^25^ (2) vaccine averted AMR economic burden, (3) vaccine averted antibiotic use, (4) sense of urgency to develop antimicrobial approaches, and (5) resistant pathogen impact on equity and social justice. The feasibility of vaccine development is being assessed for each of the 30 pathogens in scope. These pathogen-specific assessments will then be presented in the context of other available approaches to contain AMR. The overall goal of the value attribution framework is to support the prioritization of decisions and investments relating to vaccine development, introduction and use. The framework, once published, will be a platform to synthesize best available evidence for the impact of vaccines against AMR, and will highlight critical knowledge data gaps. Subsequently, this will also inform the development of a core set of evidence-based AMR interventions in the human health sector, including vaccines, that countries should consider in the revisions of their national action plans on AMR. In addition, the role of vaccines in preventing AMR will be included as a key intervention within the new “people-centered framework” for addressing AMR in the human health sector that is being developed by WHO.

To ensure vaccines are optimally utilized to reduce the emergence and spread of AMR, WHO is engaging stakeholders in policy and decision-making at global and national levels. At the global level, WHO will work closely with the Strategic Group of Experts on Immunization (SAGE) to ensure the value of vaccines against AMR is systematically considered whenever decisions relating to vaccine introduction and use are made. To drive impact at the country level, WHO is working with countries and implementing partners to consider scaling up investment in vaccines as part of their National Action Plans on AMR,^21^ and to include indicators on immunization coverage within their national action plan implementation monitoring and evaluation frameworks.^22^ The proposed indicators are based on the indicators included in the monitoring and evaluation framework of the Global Action Plan on AMR that monitor immunization coverage for pneumococcal conjugate vaccine (PCV), rotavirus vaccine, measles-containing vaccine, and *Haemophilus influenzae* type b containing vaccine (Hib).^26^ However, only a handful of national action plans on AMR appropriately include vaccines.^27^ To improve this, governance, coordination and implementation of the national action plans on AMR should be closely aligned with national immunization programs, budgets and strategies.

AMR is a complex global problem that requires multiple approaches to prevent and contain it, including vaccines. As such, WHO has outlined key strategies for vaccines to contribute fully to the prevention of AMR; evaluated the pipeline of vaccines in development for WHO priority pathogens; continues to estimate the value of vaccines in preventing AMR; and works with member states, academic institutions, non-governmental organizations, the pharmaceutical industry, and funders to better understand and communicate the impact of vaccines on AMR and implementation at country level. However, there remain challenges and opportunities for vaccines to achieve its full potential in preventing AMR. Expansion of equitable coverage to licensed vaccines should be accelerated; recommendations and guidance in vaccine and AMR sectors should include the role of vaccines in preventing AMR; awareness of the role of vaccines in preventing AMR should be increased through communication, education and training; the funding for research and development (R&D) of new vaccines needs increasing; and lastly, research to produce data on vaccine impact on AMR, especially in low-resource settings should be prioritized.
